# Role of Androgen Receptor for Reconsidering the “True” Polycystic Ovarian Morphology in PCOS

**DOI:** 10.1038/s41598-020-65890-5

**Published:** 2020-06-02

**Authors:** Xue-ying Gao, Yue Liu, Yue Lv, Tao Huang, Gang Lu, Hong-bin Liu, Shi-gang Zhao

**Affiliations:** 1 Center for Reproductive Medicine, Cheeloo College of Medicine, Shandong University, Jinan, Shandong 250012 China; 2 National Research Center for Assisted Reproductive Technology and Reproductive Genetics, Shandong University, Jinan, Shandong 250012 China; 3 Key Laboratory of Reproductive Endocrinology of Ministry of Education, Shandong University, Jinan, Shandong 250012 China; 4 School of Basic Medical Sciences, Cheeloo College of Medicine, Shandong University, Jinan, 250012 Shandong China; 5 CUHK-SDU Joint Laboratory on Reproductive Genetics, Faculty of Medicine, School of Biomedical Sciences, The Chinese University of Hong Kong, Hong Kong, China

**Keywords:** Diseases, Endocrinology

## Abstract

**Purpose:** Polycystic ovarian morphology (PCOM) is one of the key features of polycystic ovary syndrome (PCOS). The diagnosis of PCOM according to the Rotterdam criteria (≥12 antral follicles per ovary) is debated because of the high prevalence of PCOM in the general population. Androgen receptor (AR) is associated with the PCOS phenotype and might as well play a role during folliculogenesis. This study is aimed to investigate the expression of the *AR* in PCOS granulosa cells (GCs) and its relationship with the PCOM phenotype. **Methods:** 106 PCOS cases and 63 controls were included from the Center for Reproductive Medicine, Shandong University. The diagnosis of PCOS was following the Rotterdam criteria (2003). Total RNA was extracted from GCs retrieved from ovarian stimulation. The expression of *AR* was amplified by means of quantitative real-time polymerase chain reaction. **Results:** The *AR* expression was significantly decreased in PCOS cases, especially in the tPCOM subgroup (≥20 antral follicles per ovary). Correlation analyses showed that *AR* expression was significantly correlated with serum FSH levels in controls and non-tPCOM. In the tPCOM subgroup, the *AR* expression was significantly correlated with serum LH levels. Interestingly, the significance of these correlations gradually disappeared as the threshold of antral follicles increased above 24 for PCOM. **Conclusions:** *AR* was differently expressed in PCOS and especially in the tPCOM subtype. The correlation of *AR* expression with serum FSH and LH might be associated with the number of follicles in PCOM.

## Introduction

Polycystic ovary syndrome (PCOS) is a worldwide female endocrine disorder, with a prevalence up to 10–15% of reproductive-aged women leading to infertility because of anovulation, hyperandrogenism, and metabolic abnormalities^1^. Polycystic ovarian morphology (PCOM) is one of the key features of PCOS, characterized by increased recruitment of pre-antral and antral follicles but fail to progress to ovulation. Due to the observer’s variability and the improvements in imaging technology, the threshold of ≥12 antral follicles per ovary defining the diagnosis of PCOM might lead to over-diagnosis. Moreover, several studies show a high prevalence of PCOM in the population leading to a continuous debate considering the best threshold for PCOM^2–4^. The recently published international PCOS guideline recommends based on the existing evidence ≥20 follicles per ovary^5^. Up until now, molecular evidence has not been included in the discussion considering PCOM threshold^4,6^.

The majority of women with PCOS suffer from hyperandrogenism. Androgens act via the androgen receptor (AR) in a variety of tissues. *AR* mutations are found in complete androgen insensitivity patients^7^. The AR signalling pathway has been recognised as a potential factor influencing ovarian function leading to anovulation in PCOS. Studies found that the *AR* (CAG)n polymorphic trinucleotide repeats in the N-terminal domain^8^ and rs6152 gene polymorphisms^9^ are associated with PCOS. The AR exhibits distinct expression patterns at different stages of follicle growth. The AR expression is high in GCs of pre- and early antral follicles and decreases as the follicles maturate. This might indicate that AR-mediated androgens might play a role during folliculogenesis^10,11^.

There is some evidence that global *AR* knockout (ARKO) mice exhibit subfertility^12,13^. The lack of androgen activity in the GCs leads to prolonged oestrous cycle, increased number of pre-antral and atretic follicles with decreased corpora lutea and ovulation rates^14,15^. In addition, the theca cell and the oocyte-specific ARKO mice show normal fertility and follicle populations^15,16^, implying the important role of AR signalling in GCs. Studies of the expression levels of *AR* in PCOS GCs are few and the results of these studies are not consistent. The *AR* expression of GCs from small and large antral follicles from PCOS women is controversial. It is inconsistent that the *AR* expression is higher or lower in different GCs from PCOS women^17,18^. We supposed that the controversial results might be related to the different phenotypes of PCOS, due to its heterogeneity. Therefore, we studied the expression of *AR* in luteinized GCs in a large group of PCOS patients and further analyzed its relationship with PCOM phenotype.

## Material and Methods

### Study population

A total of 169 Chinese women were recruited in the Center for Reproductive Medicine, Shandong University from October 2015 to June 2016. The participants consisted of 106 PCOS cases and 63 controls. The diagnosis of PCOS was defined according to the Rotterdam criteria^19^. PCOS was diagnosed when at least two of the following criteria were present: oligo- or anovulation, clinical and/or biochemical signs of hyperandrogenism, polycystic ovaries with exclusion of other etiologies (e.g. congenital adrenal hyperplasia, androgen-secreting tumors, Cushing’s syndrome). Control women had a regular menstrual cycle (26–35 days) and steroid hormone levels within normal range. They had a normal ovarian morphology. Control women visited the IVF center because of oviduct and/or male factors related infertility.

### Clinical and biochemical measurement

All participants’ anthropometric variables, including age, height, body weight, and menstrual cycle were recorded. The levels of day 3 serum hormones including follicle stimulating hormone (FSH), luteinizing hormone (LH), oestradiol (E2), progesterone (P), total testosterone (TT) and anti-Müllerian hormone (AMH) were measured in the clinical laboratory of Center for Reproductive Medicine, Shandong University by chemiluminescence immunoassay (CLIA) and Enzyme-Linked Immuno-sorbent Assay (ELISA). Antral follicle count (AFC) was assessed by transvaginal ultrasound.

### Ovarian stimulation and granulosa cells (GCs) collection

For ovarian stimulation, the long gonadotropin-releasing hormone agonist protocol was used. All participants were injected with a gonadotropin-releasing hormone (GnRH) agonist at the beginning of the mid-luteal phase, and the ultrasound scan for follicle development and serum oestradiol assays were performed every 1 to 3 days. When more than 3 follicles measured ≥18 mm in diameter, moderate human chorionic gonadotropin (hCG) was administrated. Ultrasound-guided oocyte retrieval was performed 36 hours after hCG injection. The GCs were collected in sterile tubes from the follicular fluid and isolated with Ficoll-Percoll (Solarbio-Life-Sciences, Beijing, China) as previously described^20^.

### RNA extraction and quantitative real-time polymerase chain reaction (qRT-PCR)

Total RNA was extracted from GCs using TRIzol Reagent (Takara Bio, Inc., Dalian, China) following the manufacturer’s instructions, and was reversely transcribed to cDNA using Prime Script RT reagent Kit with gDNA Eraser (Takara Bio, Inc., Dalian, China). qRT-PCR was performed using SYBR Premix Ex Taq (Takara Bio, Inc., Dalian, China) on a LightCycler 480 system according to the manufacturer’s instructions. The primers were shown as Supplementary Table 1. The housekeeping gene *18sRNA* was used for normalization and the relative expression of *AR* mRNA was calculated based on the 2^−ΔCt^ method^21^.

### Study design and statistical analyses

The PCOS cases were grouped into non-true-PCOM (non-tPCOM, <20 AFC per ovary) and true-PCOM (tPCOM, ≥20 AFC per ovary) for preliminary data analysis based on the threshold of PCOM suggested by the recently published international PCOS guideline^5^. Based on the preliminary correlation analyses, the threshold of AFC gradually increased, and these cases were divided into PCOM subgroup. The remaining PCOS cases were defined as non-PCOM. New grouped non-PCOM and PCOM subgroups were only performed association analyses between the *AR* expression and endocrine parameters. The study group design was shown as Fig. S1.

Data were analyzed using SPSS 20.0. Data distribution was assessed using the Kolmogorov-Smirnov test to determine whether continuous variables were normally distributed. Abnormal distribution data were transformed into normal distribution data. Student’s t-test was used to determine statistical significance for baseline characteristics between PCOS cases and the controls. Two-way ANOVA followed by Bonferroni and Dunnett-T3 test was performed for multiple comparisons amongst controls, non-tPCOM and tPCOM groups. The association analyses of the *AR* expression with endocrine parameters were performed using Spearman test. *p* < 0.05 was statistically significant.

### Ethical statement

All experimental protocols performed in studies involving human participants were in accordance with the ethical standards of the Ethics Committee of Shandong University and with the 1964 Helsinki declaration and its later amendments or comparable ethical standards. Written informed consent was obtained from each patient. All experimental protocols were performed in accordance with relevant guidelines and regulations approved by the Institutional Review Board of Shandong University.

## Results

### Baseline characteristics

We collected GCs from 63 controls and 106 PCOS cases. All participants were 20 to 35 years old. The comparison of the anthropometric, biochemical and endocrine parameters between PCOS patients and controls was shown in Table 1. As expected, PCOS patients had higher BMI, serum LH, progesterone, TT and AMH levels, as well as AFC compared to the controls. Serum FSH was significantly lower (*p* < 0.05). No significant differences were found in age and serum oestradiol levels (*p* > 0.05).

**Table 1:** Anthropometric, biochemical and hormonal data of the control, PCOS and subgroups.

Clinical parameter	Control (n = 63)	PCOS (n = 106)		
		All	non-tPCOM (n = 89)	tPCOM (n = 17)
Age, year	28.79 ± 2.94	28.63 ± 3.36	28.59 ± 3.39	28.82 ± 3.33
BMI, kg/m^2^	21.79 ± 2.91	25.01 ± 4.16^*^	24.44 ± 4.12^*^	27.99 ± 3.03^*,Ɨ^
FSH, IU/L	6.70 ± 1.22	5.87 ± 1.41^*^	5.92 ± 1.43^*^	5.63 ± 1.30^*^
LH, IU/L	5.17 ± 1.66	8.65 ± 4.64^*^	8.55 ± 4.82^*^	9.18 ± 3.63^*^
E2, pg/ml	35.36 ± 10.98	38.76 ± 17.17	38.84 ± 17.53	38.38 ± 15.62
P, ng/ml	0.59 ± 0.18	0.76 ± 0.46^*^	0.78 ± 0.49^*^	0.68 ± 0.24
TT, ng/dl	23.34 ± 7.49	40.54 ± 17.45^*^	38.02 ± 16.53^*^	53.76 ± 16.54^*,Ɨ^
AMH, ng/ml	4.36 ± 2.49	9.80 ± 4.94^*^	8.93 ± 4.41^*^	14.86 ± 5.00^*,Ɨ^
FNPO	6.54 ± 1.84	13.85 ± 5.75^*^	11.85 ± 3.16^*^	24.32 ± 4.85^*,Ɨ^
Gn dose, U	1783.63 ± 813.71	1798.76 ± 887.77	1702.32 ± 774.03	2303.68 ± 1246.74

Values are expressed as mean ± standard deviation.

BMI: body mass index; FSH: follicle stimulating hormone; LH: luteinizing hormone; E2: oestradiol; P: progesterone; TT: total testosterone; AMH: anti-Müllerian hormone; FNPO: follicle numbers per ovary; Gn: gonadotropin-releasing hormone agonist.

non-tPCOM: <20 follicle numbers per ovary.

tPCOM: ≥20 follicle numbers per ovary.

^*^p < 0.05 versus Control group.

^Ɨ^*p* < 0.05 versus non-tPCOM group.

### *AR* expression in granulosa cells of controls and PCOS cases

**Figure 1:**
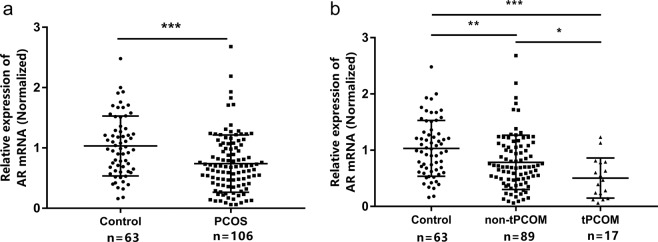
*AR* expression in GCs of controls and women with PCOS. Data were normalized by *18sRNA*. (**a**) The relative expression of *AR* mRNA in PCOS cases (n = 106) and control women (n = 63). (**b**) The normalized expression of *AR* mRNA in non-tPCOM and tPCOM subgroups. Statistical analysis of the data was performed using the non-parametric test and Two-way ANOVA followed by Bonferroni test (^*^p < 0.05, ^**^p < 0.01, ^***^p < 0.001).

qRT-PCR analysis showed that the *AR* mRNA expression in PCOS patients was significantly lower compared to the controls (*p* < 0.001; Fig. 1a). To study the relationship of *AR* with the PCOM phenotype, the PCOS cases were divided into two subgroups according to the threshold of 20 follicles per ovary (Table 1). The expression of *AR* in PCOS with tPCOM group was lower than that in control group (p < 0.001) and non-tPCOM group (p < 0.05; Fig. 1b).

### Association of the *AR* expression with clinical characteristics in subgroup

**Figure 2:**
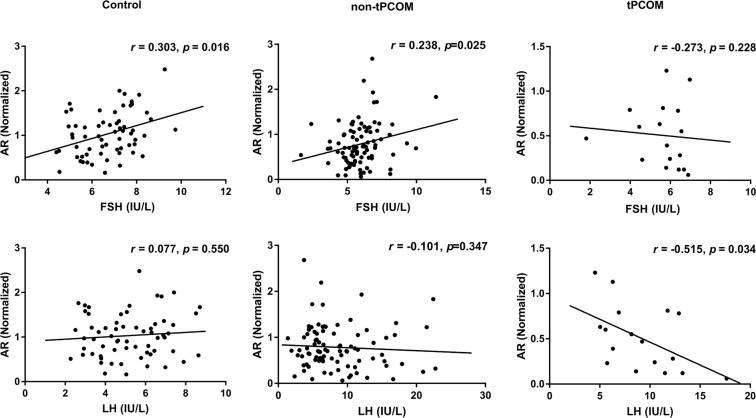
Correlation of *AR* expression with different clinical characteristics. Relationship between *AR* expression levels and serum FSH levels and serum LH levels in control group, non-tPCOM group and tPCOM group. Statistical analysis of the data was performed using Spearman test.

The correlation analyses showed that the expression of *AR* was positively correlated with serum FSH levels (*r* = 0.303; *p* = 0.016; Fig. 2) in the control group and non-tPCOM group (*r* = 0.238; *p* = 0.025; Fig. 2) but had no significant correlation in tPCOM group (*r* = −0.273; *p* = 0.228; Fig. 2). Meanwhile, the *AR* expression exhibited a negative correlation with serum LH levels (*r* = −0.515; *p* = 0.034; Fig. 2) only in tPCOM group. No correlations were found between the *AR* expression and other endocrine factors (Supplementary Table 2).

We then studied the correlations between the *AR* expression and endocrine factors with different thresholds of follicles in PCOS. The correlations between the *AR* expression and the serum FSH and LH levels had the similar significance when PCOM diagnosed by the popular criteria (≥12 antral follicles per ovary). The significant correlation between the *AR* expression and serum FSH levels was lost at the threshold of 24 follicles per ovary in non-PCOM subgroup, and with the threshold increased the correlation coefficient gradually reduced. While in PCOM group, there was no significant correlation between the *AR* expression and serum FSH levels. In the meanwhile, the relationship between the *AR* expression and serum LH levels showed non-significant in PCOM group at this threshold (Table 2; Fig. S2). No other significant changes of correlations were found in all groups (Supplementary Table 2).

**Table 2:** Spearman correlation coefficients between *AR* expression and serum FSH and LH levels in regrouped non-PCOM and PCOM subgroups.

Threshold of FNPO	non-PCOM				PCOM			
	FSH, IU/L		LH, IU/L		FSH, IU/L		LH, IU/L	
	*r*	*p*	*r*	*p*	*r*	*p*	*r*	*p*
12	0.553*	0.000	0.024	0.871	−0.099	0.454	−0.290*	0.026
20	0.238*	0.025	−0.101	0.347	−0.273	0.288	−0.515*	0.034
21	0.223*	0.030	−0.109	0.296	−0.266	0.404	−0.762*	0.004
22	0.212*	0.038	−0.136	0.188	−0.455	0.187	−0.648*	0.043
23	0.210*	0.039	−0.141	0.167	−0.500	0.170	−0.700*	0.036
24	0.193	0.055	−0.155	0.125	−0.321	0.482	−0.536	0.215
26	0.179	0.074	−0.170	0.090	0.000	1.000	−0.300	0.624

*Significant correlation as assessed by Spearman’s correlation method.

FNPO: follicle numbers per ovary; FSH: follicle stimulating hormone; LH: luteinizing hormone.

## Discussion

In the present study, we showed that the expression of *AR* was significantly lower in PCOS GCs, especially in PCOS-tPCOM subgroup, which was partly consistent with recently published studies^17,22^. The association of the *AR* expression and serum FSH levels was similar in the non-tPCOM cases and the control group. On the contrary, the tPCOM group exhibited a different pattern. The correlations between *AR* and FSH levels and LH levels were disappeared in subgroups above 24 follicles, indicating that different phenotypes of PCOS arisen. This made us reconsider the proper definition of PCOM from the molecular evidence.

The different expression patterns of the AR might play a role in folliculogenesis. Studies of ARKO mice have shown that lack of androgen activity in the GCs lead to PCOS-like ovarian dysfunction including prolonged oestrous cycle, increased number of pre-antral and atretic follicles, and a significant reduction in the number of corpora lutea as well as ovulation rates^14,15^. Previous studies showed that the *AR* expression of luteinized GCs from small antral follicles or large antral follicles from PCOS women are both controversial. It is inconsistent that the *AR* expression is higher or lower in different GCs from PCOS women. Some studies demonstrated that AR is highly expressed when induced by androgen in animal models, indicated that the highly expressed *AR* in PCOS resulted from hyperandrogenism^23^. While our present study indicated that PCOS GCs had lower *AR* expression levels, especially in patients with a greater number of antral follicles. The post-hoc analysis was done on completion of the study and the results showed that the power of our sample size was over 0.90.

Due to the ethical limits of achieving GCs from small antral follicles from the normal women, we used freshly isolated GCs without culture *in vitro* to try to be more representative of large antral follicles *in vivo*. All the participants used the similar dose of gonadotropin-releasing hormone agonist for ovarian stimulation, so the ovarian stimulation drugs were not considered as the confounding effects. As the luteinized GCs from large antral follicles obtained during IVF were not comparable to the GCs from small follicles, it was difficult to conclude that the lower *AR* expression was the pathogenesis of PCOS. We supposed that the expression of *AR* increased by short stimulation of androgen *in vivo* and *in vitro*; while the expression of *AR* might be inhibited due to the increased activity of AR in a chronic environment of hyperandrogenism like PCOS. And the PCOS women have more large antral follicles and the *AR* expression is decreased during the folliculogenesis. Thus, we suspected that decreased *AR* expression might be related to the PCOM phenotype.

We then analyzed the correlations of the *AR* expression in GCs and other factors. It showed interesting correlations with FSH and LH. It is known that FSH stimulates follicle growth moderately in synergy with other stimulating factors such as androgens. In PCOS, follicles show increased sensitivity to FSH, but because multiple follicles synchronously develop, the FSH level is relatively insufficient for each follicle. Consequently, the growth of larger antral follicles is arrested^24,25^. FSH and androgens both act via their receptors. It showed that the expression of *AR* mRNA precedes that of follicle stimulating hormone receptor (*FSHR*) in human pre-antral follicles. A positive correlation has been described previously between *AR* and *FSHR* mRNA levels in the GCs from normal cyclic, androgen or FSH-treated primates^26^. Moreover, this is described in GCs of antral follicles in PCOS^18,27^. Although exogenous androgens stimulate mRNA expression of the *FSHR* in follicles at all development stages, FSH only increases *AR* mRNA levels in primary follicles^26^. It suggests some interaction between AR and FSH in early follicular development. It has been suggested^28^ that transgenic FSH can partially rescue the subfertility phenotype and ovarian function in ARKO mice. This principle is also used in ovulation induction treatment in anovulatory women with PCOS. The positive correlation of the *AR* expression in GCs with FSH levels in controls and non-PCOM cases found in our study was in line with the previously published studies. It also indicated that there might be a larger number of antral follicles as well as atretic follicles in true PCOM cases.

In normal ovaries, only the GCs from a large (13 mm in diameter) and dominant follicle respond to LH. Cells derived from women with PCOS have inappropriate responsiveness to LH in some follicles as small as 2–4 mm. Also, high basal levels of LH show an exaggerated response in a proportion of medium-sized antral follicles^29,30^. We found the significant negative correlation between *AR* expression and serum LH levels in true PCOM cases, indicating that the interrelationship of androgen and LH might have an impact on the medium to large-sized antral follicles. Together with our previous reports^31^, which showed LHCGR is increased in PCOS GCs, the abnormal expression of these receptors might contribute to the abnormal antral follicles in PCOS.

In this study, we accidentally found that the expression of *AR* was significantly positively correlated with FSH in control and non-tPCOM groups while had a negative correlation tendency with FSH in tPCOM group when defined by 20 follicles per ovary. And the *AR* expression was significantly negatively correlated with LH in tPCOM but did not show a similar correlation in the other two groups. It made us wonder why those correlations of *AR* and endocrine levels were different in tPCOM and non-tPCOM. We suspected that these correlations might represent special features of PCOM except the ultrasound change. Hence, we increased the threshold of follicles per ovary and analyzed the correlation changes. To our surprise, we found that the significance of correlation disappeared above a follicle threshold of 24 follicles per ovary. It indicated that different phenotypes of PCOS arisen over 24 follicles per ovary, which made us reconsider the definition of PCOM.

The recently published international PCOS guideline recommends based on the existing evidence ≥20 follicles per ovary^5^. While some researches supported that ≥25 follicles per ovary might be more suitable for PCOM definition^4^. However, it is hard to define an appropriate strict threshold for PCOM. Though *AR* expression in GCs could not be considered as a diagnostic marker of PCOM, our findings of the interesting correlation pattern below and over 24 follicles might provide evidence from the molecular level that a stricter threshold might be more suitable for true PCOM.

## Conclusion

To summarize, we have investigated that the *AR* expression in GCs of PCOS patients was significantly reduced, and the reduction was much more significant in the tPCOM subgroup, indicating that the AR-mediated action might play important roles for the folliculogenesis of PCOS. The significant correlations of the *AR* expression in GCs with FSH and LH were lost above a follicle threshold of 24 follicles per ovary.

## Supplementary information

Supplementary Figure S1.

Supplementary Figure S2.

Supplementary information.

## References

[1] Norman RJ, Dewailly D, Legro RS, Hickey TE (2007). Polycystic ovary syndrome. The Lancet.

[2] Johnstone EB (2010). The polycystic ovary post-rotterdam: a common, age-dependent finding in ovulatory women without metabolic significance. The Journal of clinical endocrinology and metabolism.

[3] Bentzen JG (2013). Ovarian antral follicle subclasses and anti-mullerian hormone during normal reproductive aging. The Journal of clinical endocrinology and metabolism.

[4] Dewailly D (2014). Definition and significance of polycystic ovarian morphology: a task force report from the Androgen Excess and Polycystic Ovary Syndrome Society. Human reproduction update.

[5] Teede HJ (2018). Recommendations from the international evidence-based guideline for the assessment and management of polycystic ovary syndrome. Human reproduction.

[6] Lujan ME (2013). Updated ultrasound criteria for polycystic ovary syndrome: reliable thresholds for elevated follicle population and ovarian volume. Human reproduction.

[7] McPhaul MJ (2002). Androgen receptor mutations and androgen insensitivity. Molecular and cellular endocrinology.

[8] Shah NA (2008). Association of androgen receptor CAG repeat polymorphism and polycystic ovary syndrome. The Journal of clinical endocrinology and metabolism.

[9] Peng CY, Long XY, Lu GX (2010). Association of AR rs6152G/A gene polymorphism with susceptibility to polycystic ovary syndrome in Chinese women. Reproduction, fertility, and development.

[10] Walters KA (2015). Role of androgens in normal and pathological ovarian function. Reproduction.

[11] Astapova, O., Minor, B. M. N. & Hammes, S. R. Physiological and Pathological Androgen Actions in the Ovary. *Endocrinology* (2019). 10.1210/en.2019-00101 PMC6937455 30912811

[12] Walters KA, Simanainen U, Handelsman DJ (2010). Molecular insights into androgen actions in male and female reproductive function from androgen receptor knockout models. Human reproduction update.

[13] Walters KA, Handelsman DJ (2018). Role of androgens in the ovary. Molecular and cellular endocrinology.

[14] Walters KA (2012). Targeted loss of androgen receptor signaling in murine granulosa cells of preantral and antral follicles causes female subfertility. Biology of reproduction.

[15] Sen A, Hammes SR (2010). Granulosa cell-specific androgen receptors are critical regulators of ovarian development and function. Molecular endocrinology.

[16] Ma Y (2017). Androgen Receptor in the Ovary Theca Cells Plays a Critical Role in Androgen-Induced Reproductive Dysfunction. Endocrinology.

[17] Yang F (2015). Follicular hyperandrogenism downregulates aromatase in luteinized granulosa cells in polycystic ovary syndrome women. Reproduction.

[18] Catteau-Jonard S (2008). Anti-Mullerian hormone, its receptor, FSH receptor, and androgen receptor genes are overexpressed by granulosa cells from stimulated follicles in women with polycystic ovary syndrome. The Journal of clinical endocrinology and metabolism.

[19] Revised 2003 consensus on diagnostic criteria and long-term health risks related to polycystic ovary syndrome. *Fertility and sterility* **81**, 19–25 (2004). 10.1016/j.fertnstert.2003.10.004 14711538

[20] Li M (2019). The HMGA2-IMP2 Pathway Promotes Granulosa Cell Proliferation in Polycystic Ovary Syndrome. The Journal of clinical endocrinology and metabolism.

[21] Schmittgen TD, Livak KJ (2008). Analyzing real-time PCR data by the comparative CT method. Nature Protocols.

[22] Owens Lisa Ann, Kristensen Stine Gry, Lerner Avi, Christopoulos Georgios, Lavery Stuart, Hanyaloglu Aylin C, Hardy Kate, Yding Andersen Claus, Franks Stephen (2019). Gene Expression in Granulosa Cells From Small Antral Follicles From Women With or Without Polycystic Ovaries. The Journal of Clinical Endocrinology & Metabolism.

[23] Zhang H (2016). High-fat diets exaggerate endocrine and metabolic phenotypes in a rat model of DHEA-induced PCOS. Reproduction.

[24] Dewailly D (2016). Interactions between androgens, FSH, anti-Mullerian hormone and estradiol during folliculogenesis in the human normal and polycystic ovary. Human reproduction update.

[25] Franks S, Stark J, Hardy K (2008). Follicle dynamics and anovulation in polycystic ovary syndrome. Human reproduction update.

[26] Weil S, Vendola K, Zhou J, Bondy CA (1999). Androgen and follicle-stimulating hormone interactions in primate ovarian follicle development. The Journal of clinical endocrinology and metabolism.

[27] Nielsen ME (2011). In human granulosa cells from small antral follicles, androgen receptor mRNA and androgen levels in follicular fluid correlate with FSH receptor mRNA. Molecular human reproduction.

[28] Walters KA, Edwards MC, Jimenez M, Handelsman DJ, Allan CM (2017). Subfertility in androgen-insensitive female mice is rescued by transgenic FSH. Reproduction, fertility, and development.

[29] Willis DS (1998). Premature response to luteinizing hormone of granulosa cells from anovulatory women with polycystic ovary syndrome: relevance to mechanism of anovulation. The Journal of clinical endocrinology and metabolism.

[30] Hillier SG (2001). Gonadotropic control of ovarian follicular growth and development. Molecular and cellular endocrinology.

[31] Wang P (2014). Hypomethylation of the LH/choriogonadotropin receptor promoter region is a potential mechanism underlying susceptibility to polycystic ovary syndrome. Endocrinology.

