# Supplementary figures and images for: Role of Androgen Receptor for Reconsidering the “True” Polycystic Ovarian Morphology in PCOS
Source: Sci Rep. 2020 Jun 2;10:8993. doi: 10.1038/s41598-020-65890-5 (PMC7265442; doi:10.1038/s41598-020-65890-5)

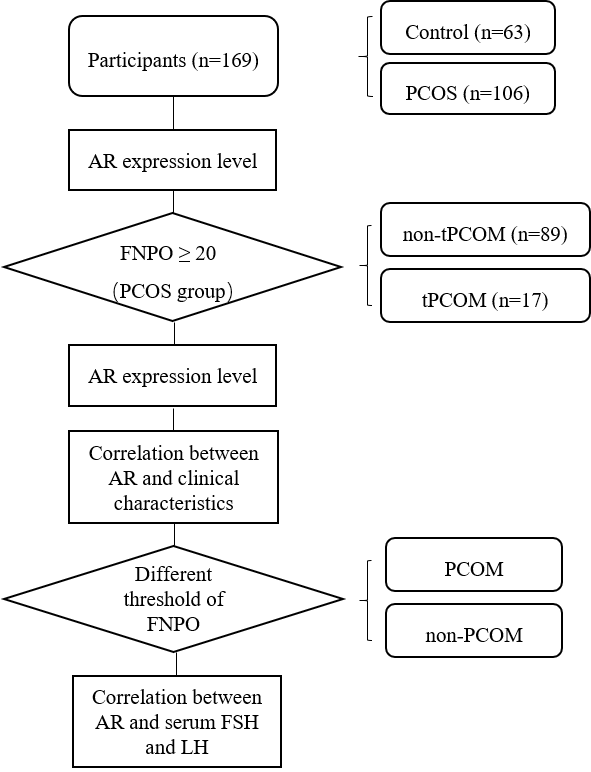

Supplement: Supplementary file 1 — Supplementary Figure S1. [file 41598_2020_65890_MOESM1_ESM.tif]

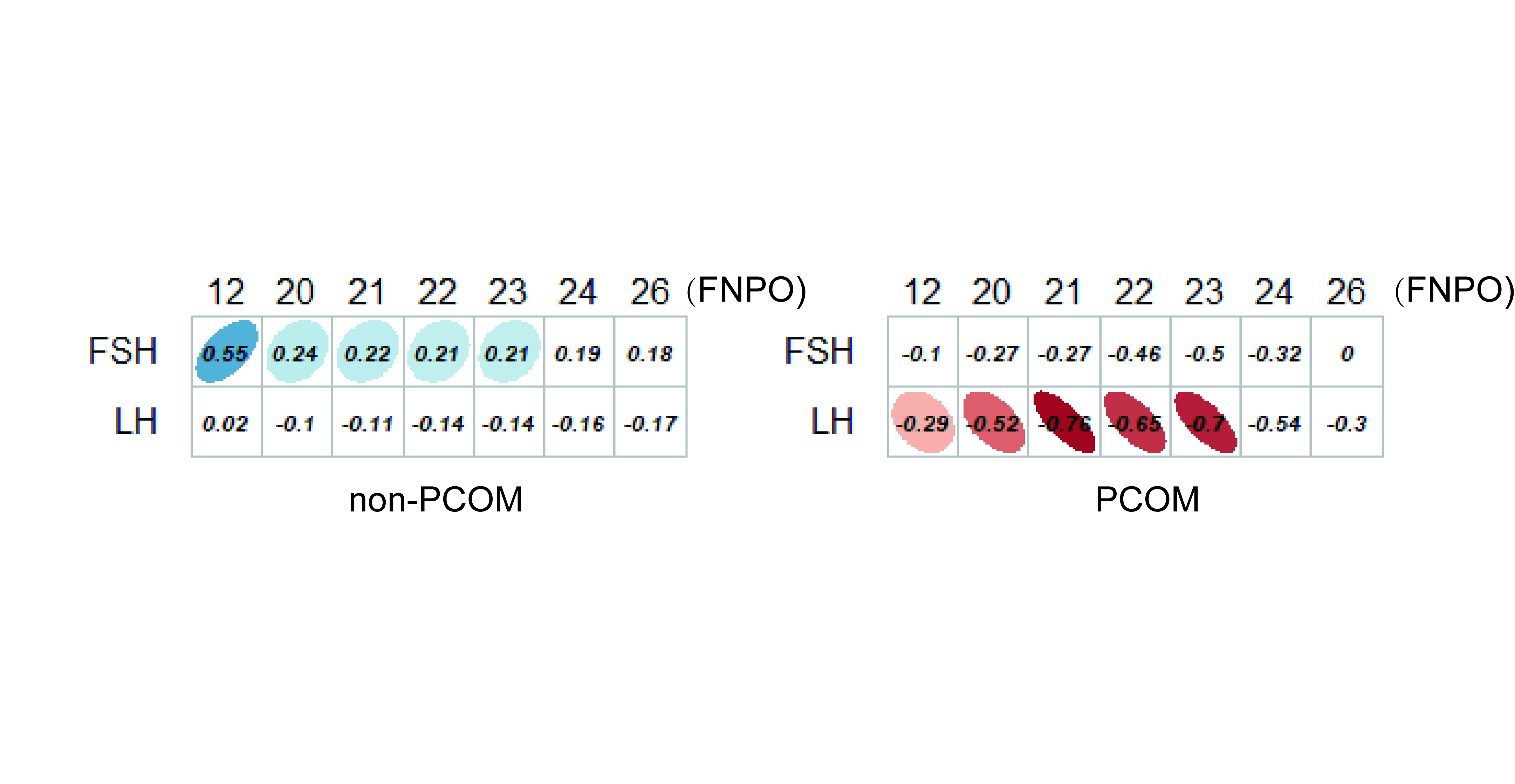

Supplement: Supplementary file 2 — Supplementary Figure S2. [file 41598_2020_65890_MOESM2_ESM.tif]
