# Supplementary material for: Role of Androgen Receptor for Reconsidering the “True” Polycystic Ovarian Morphology in PCOS
Source: Sci Rep. 2020 Jun 2;10:8993. doi: 10.1038/s41598-020-65890-5 (PMC7265442; doi:10.1038/s41598-020-65890-5)
Supplement: Supplementary file 3 — Supplementary information. [file 41598_2020_65890_MOESM3_ESM.docx]

**Role of Androgen Receptor for Reconsidering the “True” Polycystic Ovarian Morphology in PCOS**

**Xue-ying Gao^1,2,3^, Yue Liu^1,2,3^, Yue Lv^4,1,5^, Tao Huang^1,2,3^, Gang Lu^5^, Hong-bin Liu^1,2,3^, Shi-gang Zhao^1,2,3,^** ^*^

^1^ Center for Reproductive Medicine, Shandong University, Jinan 250001, China;

^2^ National Research Center for Assisted Reproductive Technology and Reproductive Genetics, Jinan 250001, China;

^3^The Key Laboratory for Reproductive Endocrinology, Shandong University, Ministry of Education, Jinan 250001, China;

^4^ School of Basic Medical Sciences, Shandong University, Jinan, China

^5^CUHK-SDU Joint Laboratory on Reproductive Genetics, Faculty of Medicine, School of Biomedical Sciences, The Chinese University of Hong Kong, Hong Kong, China.

**Corresponding author**

Shi-gang Zhao

+8618817822027

[zsg0108@126.com](mailto:zsg0108@126.com)

National Research Center for Assisted Reproductive Technology and Reproductive Genetics, No. 44, Wenhua Xi Road, Jinan 250001, People's Republic of China

**Supplementary Table 1. Primer for RT-PCR**

| Primer | Forward (5’-3’) | Reverse (5’-3’) |
| --- | --- | --- |
| *AR* | tccatcttgtcgtcttcggaa | gggctggttgttgtcgtgt |
| *18sRNA* | GGCGCCCCCTCGATGCTCTTAG | GCTCGGGCCTGCTTTGAACACTCT |

**Supplementary Table 2. Spearman correlation coefficients between *AR* expression and Age, serum E2, P, TT and AMH levels in regrouped non-PCOM and PCOM subgroups**

| Threshold of FNPO | non-PCOM | | | | | | | | | | PCOM | | | | | | | | | |
| --- | --- | --- | --- | --- | --- | --- | --- | --- | --- | --- | --- | --- | --- | --- | --- | --- | --- | --- | --- | --- |
|  | Age, year | | E2, pg/ml | | P, ng/ml | | TT, ng/dl | | AMH, ng/ml | | Age, year | | E2, pg/ml | | P, ng/ml | | TT, ng/dl | | AMH, ng/ml | |
|  | *r* | *p* | *r* | *p* | *r* | *p* | *r* | *p* | *r* | *p* | *r* | *p* | *r* | *p* | *r* | *p* | *r* | *p* | *r* | *p* |
| 12 | -0.005 | 0.975 | 0.044 | 0.768 | -0.031 | 0.837 | 0.041 | 0.784 | -0.057 | 0.705 | 0.123 | 0.353 | -0.128 | 0.335 | 0.070 | 0.607 | -0.141 | 0.286 | -0.154 | 0.257 |
| 20 | 0.014 | 0.896 | -0.085 | 0.429 | -0.021 | 0.847 | -0.019 | 0.863 | -0.103 | 0.340 | 0.406 | 0.106 | 0.077 | 0.768 | 0.136 | 0.630 | 0.043 | 0.870 | 0.111 | 0.694 |
| 21 | 0.039 | 0.708 | -0.057 | 0.589 | -0.006 | 0.956 | -0.041 | 0.695 | -0.108 | 0.306 | 0.404 | 0.192 | -0.168 | 0.602 | 0.000 | 1.000 | 0.021 | 0.948 | -0.218 | 0.519 |
| 22 | 0.063 | 0.541 | -0.059 | 0.566 | -0.006 | 0.956 | -0.049 | 0.636 | -0.124 | 0.232 | 0.311 | 0.382 | -0.224 | 0.533 | -0.067 | 0.865 | -0.030 | 0.934 | -0.033 | 0.932 |
| 23 | 0.041 | 0.687 | -0.065 | 0.527 | -0.001 | 0.992 | -0.068 | 0.505 | -0.145 | 0.160 | 0.410 | 0.273 | -0.250 | 0.516 | -0.143 | 0.736 | 0.017 | 0.966 | -0.071 | 0.867 |
| 24 | 0.052 | 0.606 | -0.048 | 0.640 | -0.014 | 0.895 | -0.081 | 0.427 | -0.171 | 0.094 | 0.393 | 0.383 | -0.464 | 0.294 | 0.200 | 0.704 | -0.107 | 0.819 | -0.029 | 0.957 |
| 26 | 0.050 | 0.620 | -0.038 | 0.706 | -0.010 | 0.922 | -0.084 | 0.401 | -0.186 | 0.066 | 0.100 | 0.873 | -0.800 | 0.104 | 0.200 | 0.747 | -0.500 | 0.391 | -0.400 | 0.600 |
| *Significant correlation as assessed by Spearman’s correlation method.  FNPO: follicle numbers per ovary. | | | | | | | | | | | | | | | | | | | | |

**
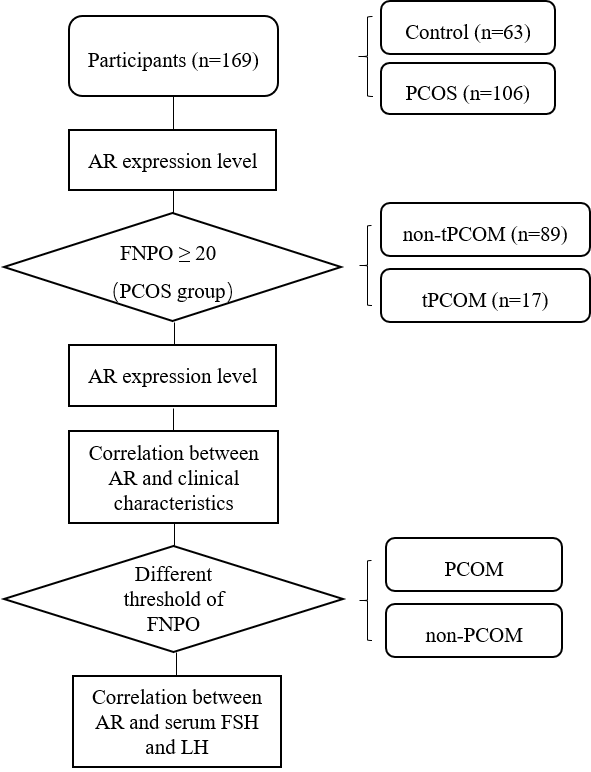
**

**Figure S1. The flow chart of the study group design.**

**
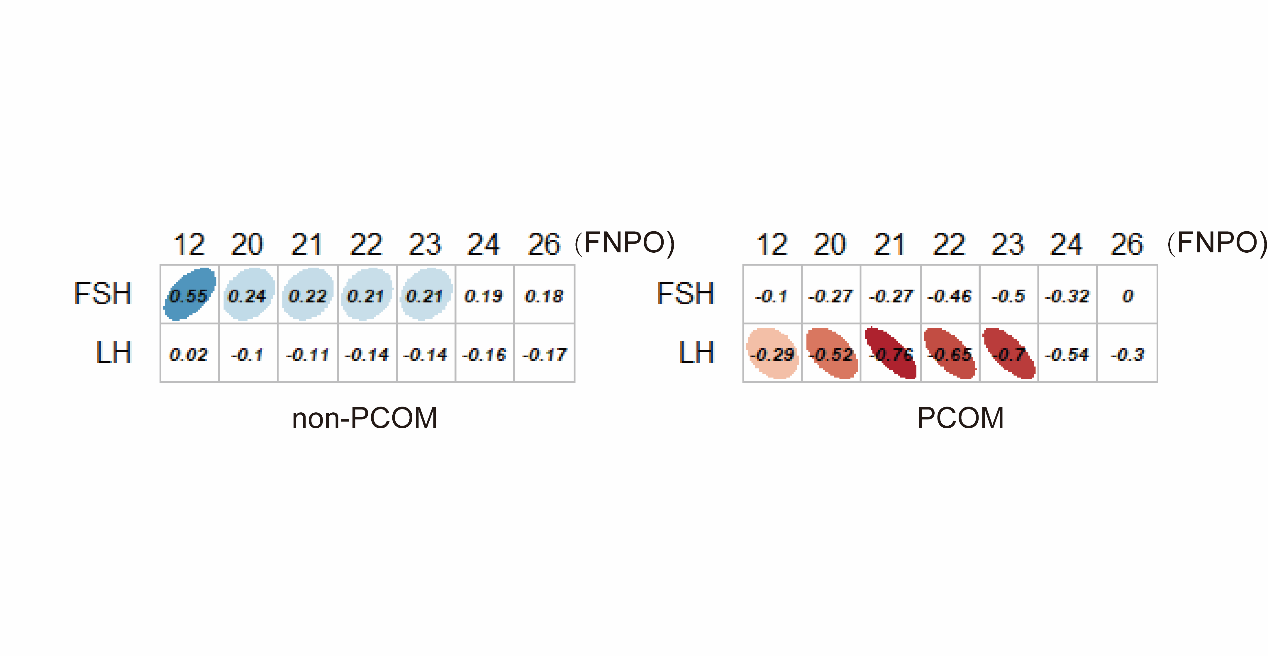
Figure S2. Correlation analysis between *AR* expression and serum FSH and LH levels in regrouped non-PCOM and PCOM subgroups.**

Relationship between *AR* expression level and serum FSH levels and serum LH levels in non-PCOM and PCOM subgroup. Statistical analysis of the data was performed using the Spearman test. The correlation coefficients were shown in numeric and only the significant correlations were shown with colored ellipses.
